# DEPRESSIVE SYMPTOMS AND SUBJECTIVE COGNITIVE FUNCTION IN OLDER ADULTS WITH MULTIMORBIDITY

**DOI:** 10.1093/geroni/igad104.1250

**Published:** 2023-12-21

**Authors:** Irina Mindlis, Tracey Revenson

**Affiliations:** Weill Cornell Medicine, New York City, New York, United States; City University of New York, New York City, New York, United States

## Abstract

Among older adults with multimorbidity (MM), depression has been associated with poorer cognitive function and vice versa; however, evidence for this temporal association is limited. Further, while the relationship between depression and cognitive function is commonly studied with dementia, less is known about the relation of depression to cognitive impairment (SCI) in older populations without dementia. We compared bidirectional models of the relationship between depressive symptoms and SCI among community-dwelling older adults with MM. Adults ≥ 62 years with MM (n = 107) recruited through a national health volunteer registry completed validated scales twice, six months apart, assessing chronic illnesses, SCI (PROMIS Cognitive Function Short Form 4a), and depressive symptoms (Geriatric Depression Scale 15). Regression analyses examined two models of depressive symptoms and SCI over time (comparing causal direction), adjusting for age, gender, and number of diseases. Participants were 63-87 years old and living with multiple illnesses (M = 4.2; range: 2-9); 21% reported moderate-to-severe depressive symptoms. Depressive symptoms at the first time point significantly predicted SCI six months later after controlling for baseline SCI (β = -.335; p < .001), such that greater depressive symptoms were associated with poorer SCI in adjusted six-month models. In comparison, SCI at baseline was not a significant predictor of depressive symptoms after controlling for initial depressive symptoms (β = -.057; p = .53) in adjusted models. Among older adults with MM, depressive symptoms may be a risk factor for future SCI or a prodromal symptom of cognitive impairment.

